# Thermosensitive and Mucoadhesive Sol-Gel Composites of Paclitaxel/Dimethyl-β-Cyclodextrin for Buccal Delivery

**DOI:** 10.1371/journal.pone.0109090

**Published:** 2014-10-02

**Authors:** Soon Gil Choi, Sang-Eun Lee, Bong-Seok Kang, Choon Lian Ng, Enkhzaya Davaa, Jeong-Sook Park

**Affiliations:** College of Pharmacy, Chungnam National University, Daejeon, Republic of Korea; Brandeis University, United States of America

## Abstract

The purpose of this study was to develop a buccal paclitaxel delivery system using the thermosensitive polymer Pluronic F127 (PF127) and the mucoadhesive polymer polyethylene oxide (PEO). The anticancer agent paclitaxel is usually used to treat ovarian, breast, and non-small-cell lung cancer. To improve its aqueous solubility, paclitaxel was incorporated into an inclusion complex with (2,6-di-*O*-methyl)-β-cyclodextrin (DMβCD). The formation of the paclitaxel inclusion complex was evaluated using various techniques, including x-ray diffractometry (XRD), Fourier-transform infrared (FT-IR) spectrophotometry, differential scanning calorimetry (DSC), and scanning electron microscopy (SEM). Hydrogels were prepared using a cold method. Concentrations of 18, 20, and 23% (w/v) PF127 were dissolved in distilled water including paclitaxel and stored overnight in a refrigerator at 4°C. PEO was added at concentrations of 0.1, 0.2, 0.4, 0.8, and 1% (w/v). Each formulation included paclitaxel (0.5 mg/mL). The sol-gel transition temperature of the hydrogels was measured using the tube-inverting method. Drug release from the hydrogels was measured using a Franz diffusion cell containing pH 7.4 phosphate-buffered solution (PBS) buffer at 37°C. The cytotoxicity of each formulation was measured using the MTT assay with a human oral cancer cell (KB cell). The sol-gel transition temperature of the hydrogel decreased when PF127 was present and varied according to the presence of mucoadhesive polymers. The *in vitro* release was sustained and the release rate was slowed by the addition of the mucoadhesive polymer. The cytotoxicity of the blank formulation was low, although the drug-loaded hydrogel showed acceptable cytotoxicity. The results of our study suggest that the combination of a PF 127-based mucoadhesive hydrogel formulation and inclusion complexes improves the *in vitro* release and cytotoxic effect of paclitaxel.

## Introduction

Paclitaxel is one of the most successful anticancer drugs discovered in the past few decades. It has been used clinically in the treatment of a wide variety of cancers including breast, ovarian, skin and lung cancer [1]. However, paclitaxel is poorly soluble in aqueous liquids due to its highly lipophilic properties [2]. For clinical administration, paclitaxel is solubilized in a mixture of ethanol and Cremophor EL (polyethoxylated castor oil), and causes serious side effects including neurotoxicity, hypersensitivity reactions and nephrotoxicity [3]–[4]. Enhancing the solubility of paclitaxel has received significant attention, as it is important to reduce the adverse effects on the patient.

To overcome the problems caused by Cremophor EL and to improve the efficacy of the drug, recent research has focused on developing new drug delivery systems, including hydrogels [5], liposomes [6], niosomes [7], nanoemulsions [8], cyclodextrin complexes [9], lipid nanocapsules [10], and polymeric micelles [11]. Inclusion complexes are frequently prepared using cyclodextrin (CD), a cyclic oligosaccharide with six to eight glucose units bonded by an α-1,4-linkage. Due to the cavity in this molecule, it is used to form inclusion complexes with many compounds, which prevents the oxidation of oils and volatilization of volatile flavors, and solubilizes insoluble compounds. It is used in the pharmaceutical field to form inclusion complexes with drug molecules to increase their aqueous solubility and stability [12]–[13], to enhance the water solubility and hydrolytic stability of curcumin [14], to improve photochemical and thermal stability [15], to mask unwanted characteristics, or to reduce side effects.

The possibility of delivering paclitaxel through the oral mucosa for local delivery using a thermoreversible mucoadhesive oral hydrogel which provides a high local concentration of drug and prevents the systemic side effects normally associated with intravenous administration is promising. A mucoadhesive drug delivery system that utilizes the bioadhesive properties of certain polymers would allow the targeting of a drug to a particular region of the body [16]–[17]. Mucoadhesive systems have advantages when compared to conventional dosage forms. First, mucoadhesive systems are readily localized, which improves the bioavailability of drugs. Also, these dosage forms facilitate intimate contact of the formulation with the underlying absorption surface. This allows modification of tissue permeability to promote the absorption of macromolecules, such as peptides and proteins.

Pluronic F127 (Poloxamer 407, PF127) is suitable for use in the formulation of topical dosage forms, because it has low toxicity and forms a thermoreversible and mucoadhesive hydrogel in aqueous media. Poloxamer consists of hydrophilic ethylene oxide (EO) and hydrophobic propylene oxide (PO) blocks, arranged in a basic structure of EO_a_-PO_b_-EO_a_. It has been widely exploited as an in-situ forming drug delivery carrier because it exhibits unique sol-gel transition behavior in response to temperature in aqueous solution [17]–[18]. These copolymers form spherical micelles in aqueous solution by means of hydrophobic interactions among the middle PPO segments. Above a critical gelation temperature and concentration, the self-assembled micelles pack closely to produce a physically cross-linked gel structure. Polyethylene oxide (PEO), a mucoadhesive polymer, not only improves the physical properties of the formulation, but also increases the surface interaction with the tissue and, consequently the contact time. Therefore, when applied onto the skin or injected into a body cavity, the gel preparation forms a solid artificial barrier and a sustained release depot [19].

In this study, we aimed to improve the solubility of paclitaxel using cyclodextrins and to prepare a mucoadhesive and thermoreversible hydrogel formulation for oral topical delivery using PF127 and PEO. The paclitaxel inclusion complex was characterized by assessing its solubility, Fourier-transform infrared (FT-IR) spectrometry, differential scanning calorimetry (DSC), and scanning electron microscopy (SEM). The mucoadhesive polymer containing paclitaxel was investigated in terms of sol-gel transition, *in vitro* release, and cell viability.

## Materials and Methods

### Materials

Paclitaxel was obtained from the Samyang Holdings Corporation (Daejeon, Republic of Korea). Acetonitrile was purchased from Burdick and Jackson (HPLC grade). HPαCD, HPβCD, HPγCD, DMβCD, PF127, PEO, sodium phosphate, potassium chloride, potassium phosphate monobasic, sodium phosphate dibasic, dimethyl sulfoxide (DMSO) and 3-(4,5-dimethylthiazol-2-yl)-2,5-diphenyltetrazolium bromide (MTT) were purchased from the Sigma Chemical Company (St. Louis, MO). The human nasopharyngeal cancer cell line (KB cell) was maintained in RPMI medium with fetal bovine serum (FBS) [17], [20]. All other chemicals and reagents used were of analytical grade.

### Phase solubility diagram

An excess amount of paclitaxel was added to an aqueous solution containing varying concentrations of HPαCD, HPβCD, HPγCD, DMβCD, and PF127. The suspensions were mixed well and shaken for 72 h at 25°C by the method of Kim et al. [16]. After equilibrium was reached, the mixtures were withdrawn and filtered through a 0.45 µm PVDF membrane filter and diluted appropriately with methanol. The filtrate was analyzed by HPLC (mobile phase, acetonitrile∶water = 50∶50; flow rate, 1.0 mL/min; injection volume, 20 µL; wavelength, UV 227 nm). The concentration of paclitaxel was calculated from the peak area of the standard curve using identical HPLC conditions. The phase solubility diagram was constructed by plotting the total dissolved drug concentration.

### Preparation of the paclitaxel/DMβCD complex

The inclusion complex of paclitaxel with DMβCD was prepared using the co-lyophilization technique [21]. One gram of DMβCD was added slowly to 10 mL of distilled water with agitation. The mixture was stirred for ∼5 min until DMβCD was completely dissolved. Paclitaxel (10 mg) was added to the DMβCD solution with vigorous stirring over a 30-min period. The dispersion was left to equilibrate for 72 h at room temperature under constant stirring. The resulting solution was filtered (0.45 µm pore size) and then lyophilized. The solution was separated into 10-mL aliquots and placed into 50-mL Falcon tubes and lyophilized overnight in a freeze dryer to obtain the complex in dry powder form.

### Physicochemical characterization of the paclitaxel/DMβCD complex

The paclitaxel/DMβCD complex was physicochemically characterized by XRD, FT-IR spectrometry, DSC, and SEM.

The crystallinity changes of the samples were observed using a powder x-ray diffractometer (Generator: D/Max-2200, Goniometer: *θ*/*θ* goniometer, Rigaku, Japan) with Ni-filtered Cu-Kα radiation, and voltage diffraction. All samples were in the 2*θ* angle range of 5° to 50° with a scan rate of 3°/min. The XRD patterns of paclitaxel as a raw material, DMβCD, and physical mixtures as well as of inclusion complexes, were recorded.

The FT-IR spectra of paclitaxel, DMβCD, physical mixture and the paclitaxel/DMβCD complex were obtained using an ATR FT-IR spectrometer (Travel IR, Sens IR Technologies, CT, USA) equipped with a DTGS detector. A resolution of 2 cm^−1^ was used, and 64 scans were co-added for each spectrum at frequencies range of 4000 to 500 cm^−1^.

The formation of the inclusion complex between paclitaxel and DMβCD was confirmed by DSC (S-650 Scinco, Ltd, Korea). Samples (2 mg) were heated in a hermetically sealed aluminum pan at a rate of 10°C/min to 300°C in a dynamic nitrogen atmosphere. All samples were analyzed in duplicate.

The morphology was determined using a scanning electron microscope (JEOL, JSM-7000F, Japan). Prior to examination, the samples were fixed on an SEM-stub using conductive double-sided tape and then made electrically conductive by coating in a vacuum with a thin layer of gold/palladium. They were then examined using a SEM at 10-kV acceleration voltage.

### Preparation of paclitaxel/DMβCD gel formulations

Paclitaxel-loaded thermosensitive gel formulations were prepared according to a previous method [16]. The paclitaxel/DMβCD complex powder was dissolved in distilled water at room temperature and cooled to 4°C. The thermosensitive polymer PF127 and the mucoadhesive polymer PEO were added to the paclitaxel/DMβCD solution at various concentrations. The liquid solution was stored at 4°C until it clarified. PF127 was used at 18, 20, and 23% w/v. PEO was added at concentrations of 0.1, 0.2, 0.4 and 0.8% to PF127 solution containing the paclitaxel/DMβCD complex. Each formulation included paclitaxel at 500 µg/mL.

### Measurement of gelation temperature

One milliliter of each polymer solution was transferred to a 4-mL vial, and stored at 4°C overnight. The sol-gel and gel-sol transitions of the polymer solutions were determined by the tube inversion method [16] in the temperature range 15–80°C with increments of 1°C per step. Each formulation was heated in a water bath for several minutes and the gelation temperature was recorded as the temperature at which a polymer solution stopped flowing after a tube inversion.

### In vitro drug release study

*In vitro* release experiments were performed at 37°C over a 10-day period. Drug release from the hydrogel was investigated using a Franz diffusion cell. Samples were injected into a donor chamber displaying a permeation area of 0.785 cm^2^, and 12.5 mL of phosphate buffered saline (PBS) pH 7.4 was added to the acceptor chamber. A dialysis membrane was placed between the donor and acceptor chambers. After a 15-min pre-incubation time to heat the acceptor chamber to 37°C, 0.2 or 0.5 mg of paclitaxel in the various formulations was placed in the donor chamber. At predetermined time points (from 1 h to 10 days) a 1-mL of sample was withdrawn from the acceptor compartment, and replaced by PBS (pH 7.4). The concentration of paclitaxel in withdrawn samples was analyzed using a Shimadzu UV-VIS 160 spectrophotometer (227 nm). The quantity of paclitaxel released from the formulation was calculated using a calibration curve obtained using an analytically validated method (r^2^ = 0.998).

### Cytotoxicity assay

The cytotoxicity of the paclitaxel gels was evaluated by MTT assay. KB cells (human oral cancer cells) were seeded at a density of 1×10^4^ cells/well in a 96-well plate overnight. Drug-unloaded and paclitaxel gels (100%, 50%, and 25%) were added to the wells and incubated overnight. After incubation, 100 µL of 5 mg/mL MTT in RPMI medium were added to each well, followed by incubation for 4 h at 37°C. To dissolve the formazan crystal, 100 µL of MTT solubilization solution were added. After shaking the 96-well plate for 30 min, the absorbance at 227 nm was measured using a micro-plate reader (Sunrise, TECAN, Switzerland). The OD of untreated cells was taken as the 100% value for determination of cytotoxicity.

### Data treatment

Data are presented as means ± standard deviation (SD). An unpaired Student's t-test was used to analyze the cumulative release data.

## Results and Discussion

### Solubility of paclitaxel

**Figure 1:**
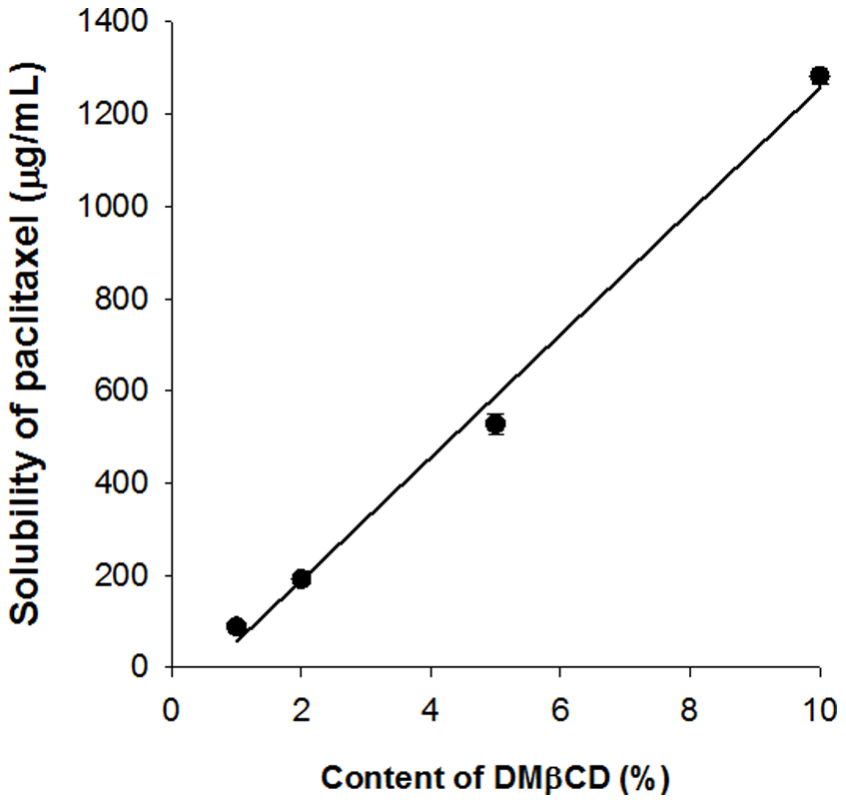
Solubility of paclitaxel by complexation with DMβCD. Each data point is the mean±SD (n = 3).

Cyclodextrin derivatives have been used widely to improve paclitaxel solubility through formation of inclusion complexes [20]–[25]. To identify a paclitaxel solubility enhancer, the effects of cyclodextrin derivatives (HPαCD, HPβCD, HPγCD and DMβCD) on paclitaxel solubility were determined (**Table 1**). Paclitaxel showed the highest solubility with DMβCD, 88.04±8.14 µg/mL. The solubility of paclitaxel increased with increasing DMβCD concentration (**Table 2**). Concentrate of paclitaxel is available in 6 mg/mL of paclitaxel and must be diluted prior to infusion [26]. It should be diluted in 0.9% sodium chloride injection, 5% dextrose injection, 5% dextrose in Ringer's injection to a final concentration of 0.3 to 1.2 mg/mL. Using the solubility diagram of paclitaxel shown in **Fig. 1**, to produce 1.2 mg/mL paclitaxel solution, 10% (w/v) DMβCD is required. Therefore, DMβCD can be considered a strong solubilizer of paclitaxel (1282.99±17.34 µg/mL with 10% (w/v) DMβCD), being superior in this respect to Cremophor EL and ethanol mixtures. Thus, a paclitaxel/DMβCD weight ratio of 1/100 was used in further studies.

**Table 1:** Effect of cyclodextrin derivatives on the solubility of paclitaxel (n = 3).

Cyclodextrin	Solubility (µg/mL)
None	0.68±0.14
HPαCD	4.58±0.85
HPβCD	15.35±2.58
HPγCD	8.35±3.12
DMβCD	88.04±8.14

The concentration of cyclodextrin derivatives is 1% (W/V).

**Table 2:** Effect of DMβCD content on the solubility of paclitaxel (n = 3).

DMβCD (%, w/v)	Solubility (µg/mL)
1	88.04±8.14
2	191.17±5.07
5	528.44±21.24
10	1282.99±17.34

The effect of PF127 on the solubility of paclitaxel was investigated (**Table 3**). A PF127 content>10% increased the solubility of paclitaxel considerably; however, <5% PF127 had no such effect. It may resulted from not enough PF127 contents to form micelles for the solubilization of paclitaxel because thermosensitive gel-forming PF127 could function as a good surfactant.

**Table 3:** Solubilizing effect of PF127 content on paclitaxel (n = 3).

PF127 (%, w/v)	Solubility (µg/mL)
1	5.05±2.51
2	8.16±1.16
5	8.72±.59
10	31.46±18.35
20	92.32±20.77

### Physicochemical characterization of inclusion complex

**Figure 2:**
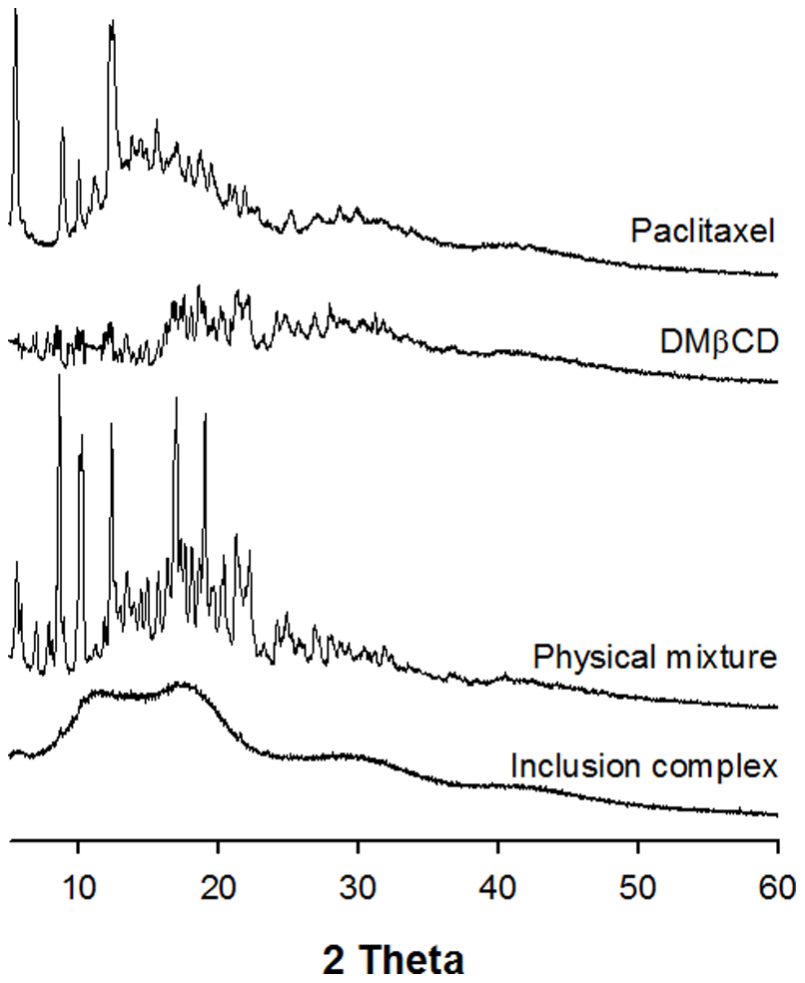
X-ray diffraction patterns of paclitaxel, DMβCD, physical mixture, and inclusion complex.

The formation of an inclusion complex was evaluated by FT-IR spectrophotometry, XRD, DSC, and SEM. Physical mixtures (1∶100 weight ratio) were prepared by simple dry mixing of paclitaxel and DMβCD. **Fig. 2** shows the FT-IR spectra of paclitaxel, DMβCD, the physical mixture, and the inclusion complex. The FT-IR spectrum for the complex was almost identical to that of DMβCD. The spectrum of paclitaxel shows strong absorption bands in the range 1700–1720 cm^−1^ (C = O), and DMβCD shows strong absorption bands in the range 3350–3500 cm^−1^ (O-H). Physical mixtures show peaks in the range 1700–1720 cm^−1^ (C = O) due to the presence of paclitaxel. In the case of the inclusion complex, the carbonyl-stretching band of the drug disappeared, suggesting that the C = O group of paclitaxel is involved in the inclusion complex.

**Figure 3:**
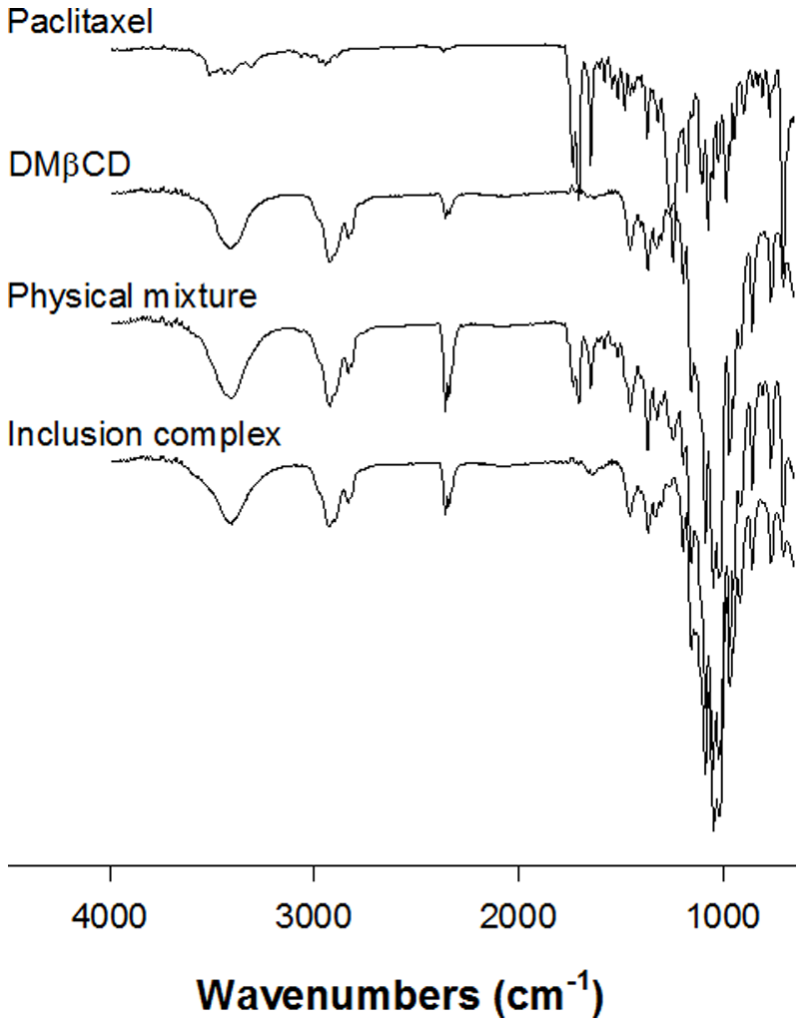
FT-IR spectra of paclitaxel, DMβCD, physical mixture, and inclusion complex.

Because changes in crystallinity are indicators of the formation of an inclusion complex, the powder x-ray diffraction patterns of paclitaxel, DMβCD, the physical mixture, and the inclusion complex were observed [16]. Paclitaxel showed a sharp peak at a diffraction angle 2*θ* 5.39, 8.97, 12.58° (**Fig. 3**), suggesting that the drug was present as a crystalline material. DMβCD also showed a diffractogram consistent with its crystalline nature. The physical mixture of paclitaxel and DMβCD exhibited a diffractogram that could be characterized as a superimposition of crystalline paclitaxel and DMβCD. In contrast, the inclusion complex diffractogram was amorphous and characterized only by large diffraction peaks in which the characteristic peaks of paclitaxel were absent. These results confirm that paclitaxel is not present as a crystalline material in its inclusion complex with DMβCD.

**Figure 4:**
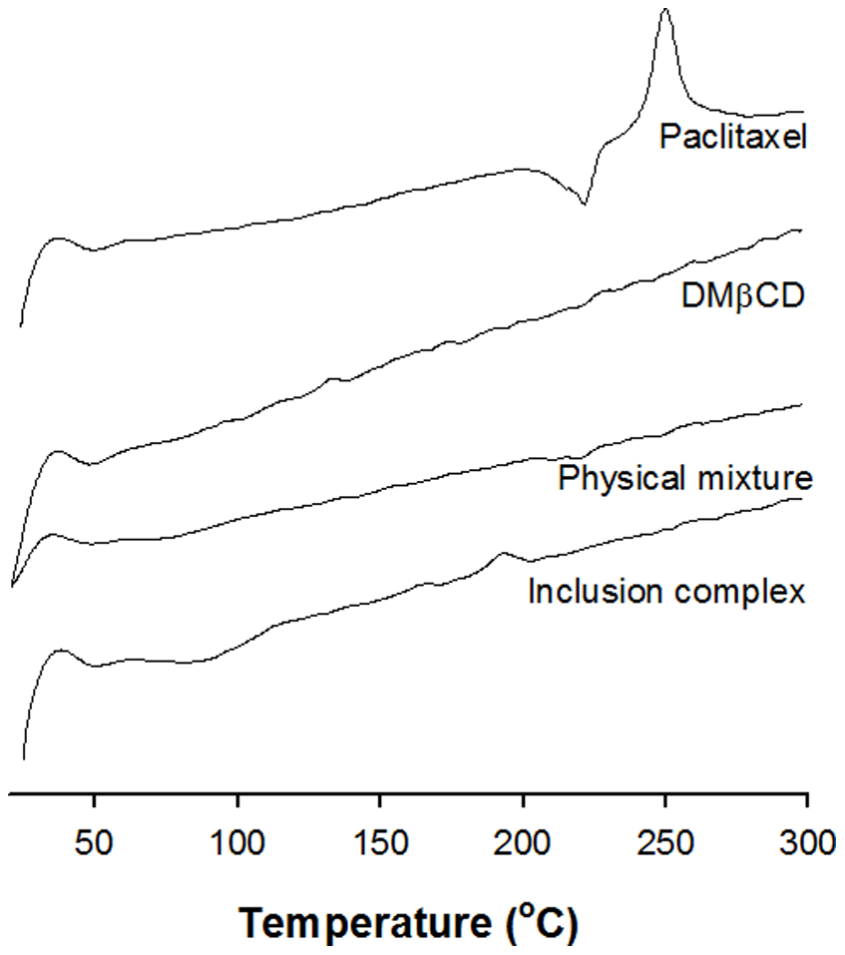
DSC curves of paclitaxel, DMβCD, physical mixture, and inclusion complex.

The DSC thermograms of paclitaxel, DMβCD, the physical mixture and the inclusion complex are shown in **Fig. 4**. The thermogram of paclitaxel exhibited a single endotherm peak at 220°C just prior to an exothermic degradation peak. In contrast, the DSC curves of DMβCD showed no peaks in the region 60–300°C. The DSC curves of the physical mixture showed a minor peak at 220°C, corresponding to the melting of paclitaxel. Disappearance or decrease in intensity of endothermic peak at 220°C and exothermic peak at 250°C of the paclitaxel and DMβCD physical mixture might be related to possible drug-cyclodextrin interactions [27] or thermal conversion of paclitaxel increased by DMβCD [28]. However, the endothermic peak corresponding to paclitaxel was absent from the inclusion complex, indicating the formation of an amorphous inclusion complex; i.e., the molecular encapsulation of the drug within the DMβCD cavity.

**Figure 5:**
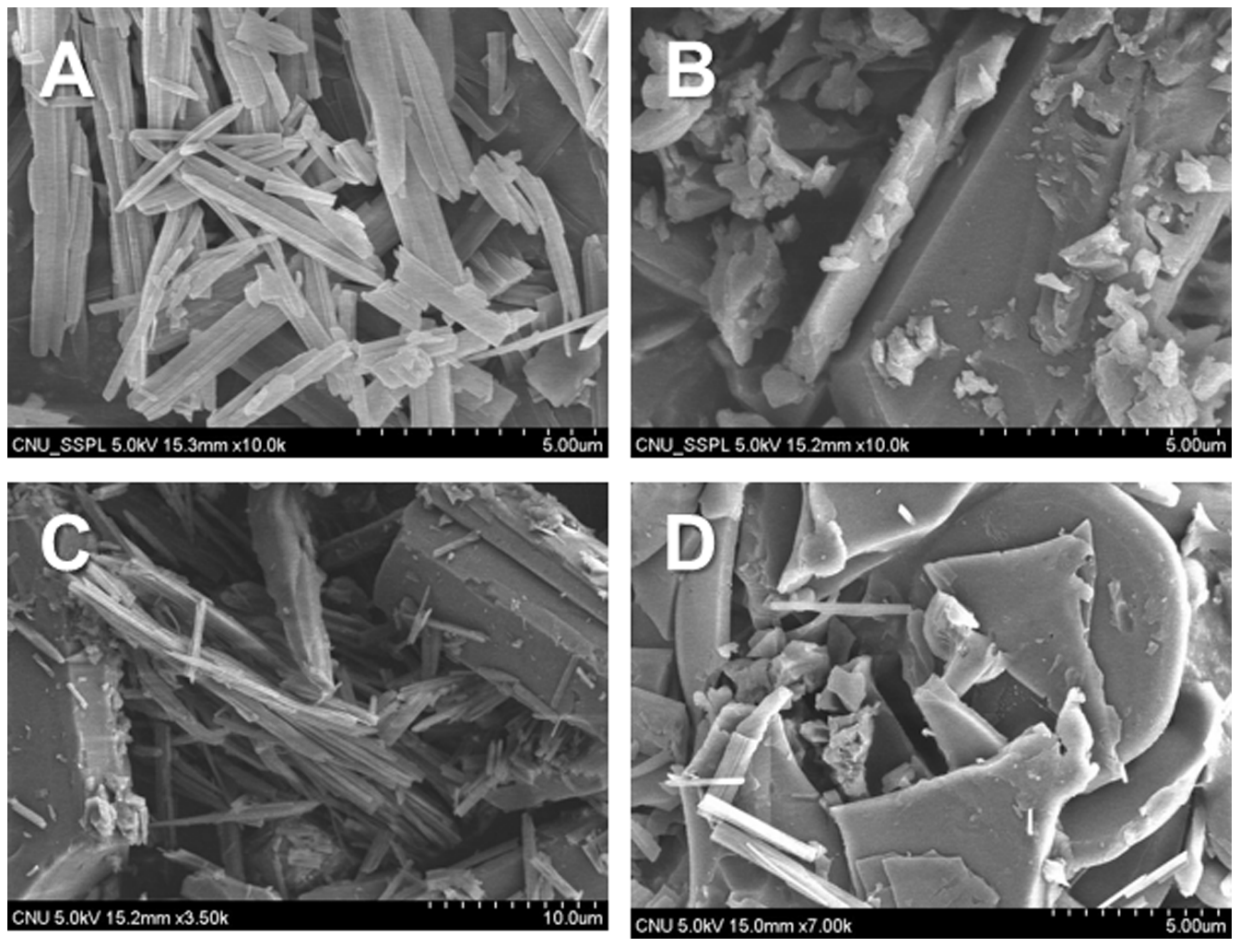
SEM images of paclitaxel (A), DMβCD (B), physical mixture (C), and inclusion complex (D).

The SEMs of paclitaxel, DMβCD, the physical mixture, and the inclusion complex are shown in **Fig. 5**. The apparent conformation of the inclusion complex was distinct from that of the isolated component. As can be seen in **Fig. 5**, paclitaxel can be characterized as a sticklike morphology, and DMβCD as plate-like particles. The physical mixtures showed particles of DMβCD embedded with paclitaxel particles. In contrast, a drastic change in the morphology and shape of particles was seen in the inclusion complex. Therefore, an inclusion complex between paclitaxel and DMβCD was formed, which contributed to the improved solubility of paclitaxel.

### Gelation temperature of the formulations

**Figure 6:**
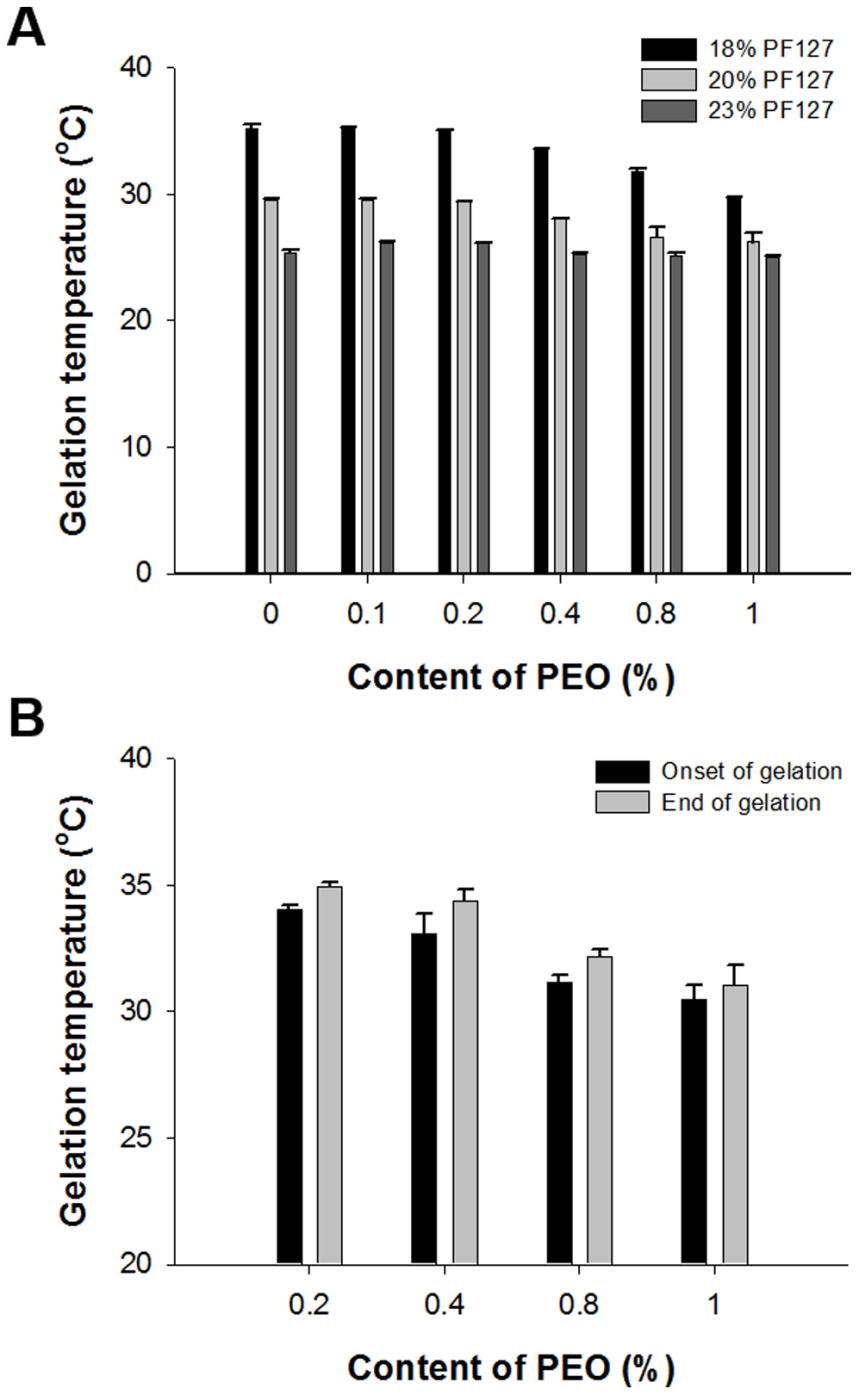
Sol-gel transition temperature of blank gel (A) and drug-loaded 20% PF 127 gel (B) formulations (n = 3).

**Fig. 6a** shows the gelation temperatures of the drug-unloading formulations. Concentrations of PF127 were 18%, 20%, and 23%; and the additional PEO contents were 0.1%, 0.2%, 0.4%, 0.8%, and 1%. Thus the gelation temperatures of the formulations decreased with increasing PF127 concentration [17]. Self-assembled micelles, formed by hydrophobic interactions of the PPO segments of PF127 in aqueous solution pack closely above the critical gelation temperature; therefore, as more PF127 was added, the gelation temperature decreased. The gelation temperatures decreased in the presence of PEO in the 18% and 20% PF127 formulations, but there was no obvious difference in the 23% PF127 formulation. It is estimated that 23% PF127 in the gelling system is relatively high; therefore a low concentration of PEO may not affect the gelation temperature.

**Fig. 6b** shows the gelation temperature of the drug-loaded gel formulations containing 20% PF127, generated by subdividing the onset and end of gelation. The sol-gel transition temperature of the drug-loaded gel formulation was slightly higher than that of the blank gel formulation. A possible mechanism of the increase of gelation temperature, is related to the methyl group (-CH_3_) of DMβCD. Due to interactions between DMβCD and PF127, the activity of PPO segments in PF127 was reduced and the critical gelation temperature was increased. Thermal transition studies showed that the aqueous solutions of the polymers gelled at body temperature and that the gelation temperature of the polymer solution was dependent on the polymer concentration. The presence of the gel phase around body temperature (37°C) indicates that the DMβCD/PF127 gel formulation shows promise as preparation for an oral drug delivery system that can be fabricated at room temperature and would form a gel *in situ* upon oral mucous administration [16].

### In vitro drug release

**Figure 7:**
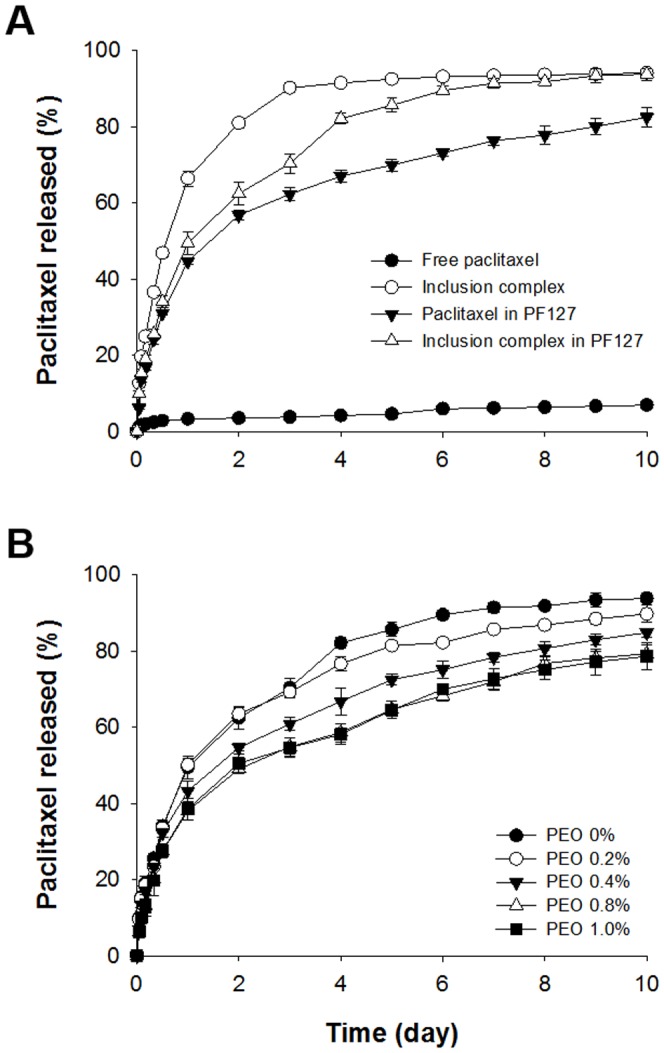
*In vitro* release of paclitaxel as free drug, complex solution, paclitaxel in PF127, inclusion complex in PF127 (A) (n = 3). The total amount of paclitaxel was 0.2 mg. Effect of PEO on the *in vitro* release of paclitaxel from inclusion complexes formulated in 20% PF127 and additional PEO (B) (n = 3). The total amount of paclitaxel was 0.5 mg.

*In vitro* release experiments were performed at 37°C for 10 days. **Fig. 7a** shows the amount of paclitaxel released from free drug, the inclusion complex solution, paclitaxel (paclitaxel powder) in PF127, and the inclusion complex (paclitaxel/DMβCD) in PF127. The amount of paclitaxel loaded was 0.2 mg. With the exception of free drug, the concentration of paclitaxel used was 0.1 mg/mL. As shown in **Fig. 7a**, little free drug was released during the 10-day period due to the poor solubility of paclitaxel in water. In contrast, the inclusion complex solution released over 90% of the drug over 3-day period; moreover, the release was sustained. The release rate was lower for the PF127-based hydrogels (paclitaxel in PF127 and inclusion complex in PF127). During the release study, some portion of hydrogel might be degraded down to small size micelles, which enhance the drug release and bioavailability [29], [30].

The amount of drug released by formulations containing the inclusion complex, PF127, and PEO was also assessed. The drug loading concentration of the inclusion complex was 0.5 mg/mL, that of polymer PF127 was 20% (w/v), and of PEO were 0.2, 0.4, 0.8, and 1% (w/v), which were used based on a previous results [29]. The addition of PEO to the inclusion complex in the 20% PF127 hydrogel formulations prolonged drug release (**Fig. 7b**). Drug release from the formulation without PEO approached 90% at 6 days; however, less than 70% of the drug was released from the 0.8% and 1% PEO formulation. Therefore, the constant release rate suggests a sufficient and prolonged anticancer effect.

### Cytotoxicity assay

**Figure 8:**
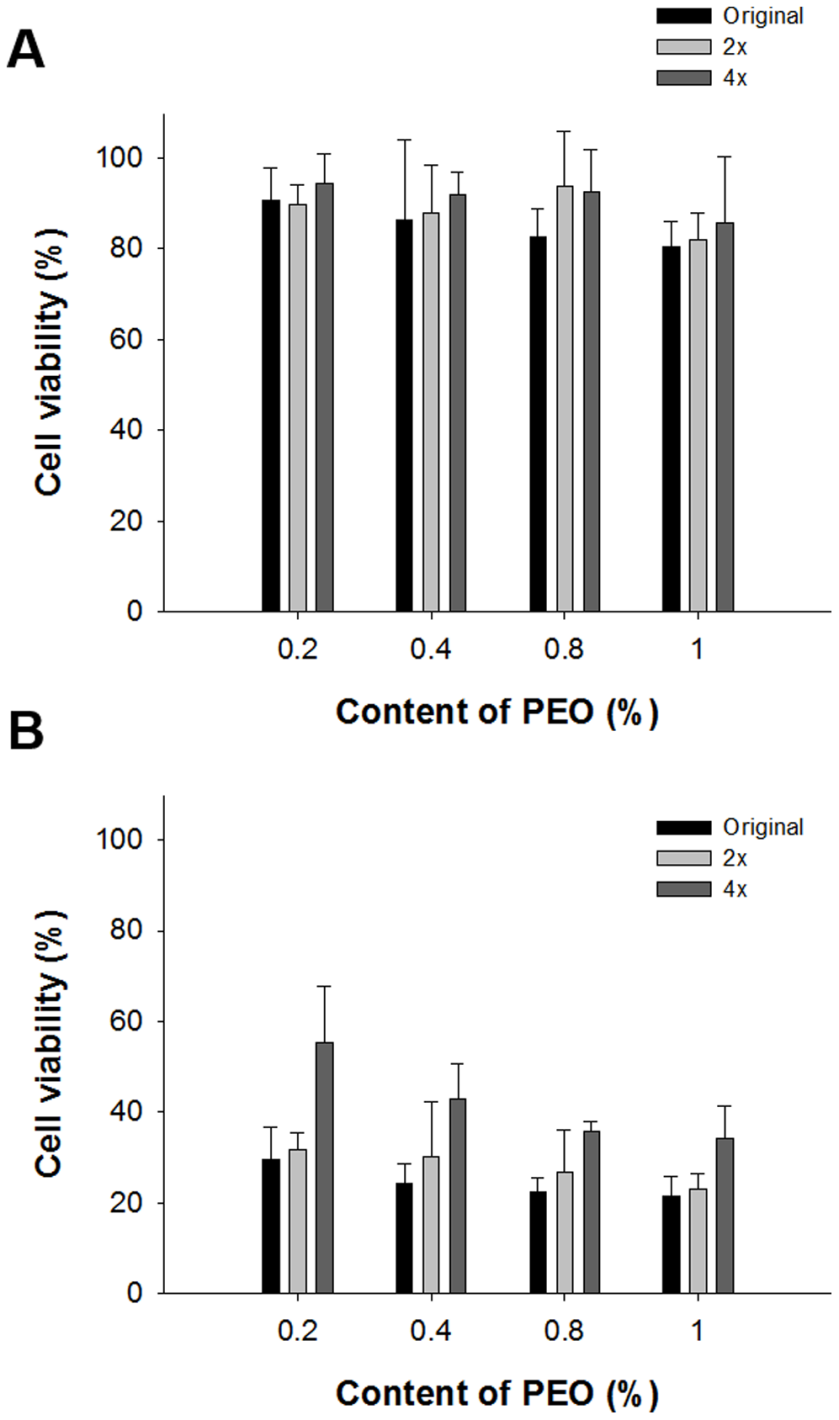
Cell viability of blank (A) and drug-loaded (B) hydrogels (n = 5).

The cytotoxicity of the inclusion complex-loaded gels was evaluated by a MTT assay using KB cells [17], [20]. Cell viabilities (%) of the blank gel and drug-loaded gel formulations are shown in **Fig. 8**. For comparison with the undiluted original formulation, drug-unloaded gels and drug-loaded inclusion complex gels were diluted with water (2×, 4×). The lowest cell viability was >80% for the drug-unloaded hydrogels, suggesting that these were not cytotoxic to KB cells (**Fig. 8a**). However, the cell viability of drug-loaded hydrogels was considerably lower due to the intact cytotoxicity of paclitaxel (**Fig. 8b**). Moreover, cell viability tended to decrease with increasing PEO concentration, albeit not significantly so. Also, dilution of the hydrogels resulted in increased cell viability, again in a non-significant manner.

## Conclusions

A thermosensitive and mucoadhesive gel formulation of a paclitaxel/DMβCD inclusion complex was prepared for oral mucosal administration using the multiblock copolymer PF127 and PEO. The paclitaxel and DMβCD inclusion complex that formed resulted in improved paclitaxel solubility. The paclitaxel gel underwent gelation at body temperature. The gelation temperature of the formulations was decreased by the addition of PF127 and PEO. *In vitro* release was sustained by the addition of PEO. Our results suggest that an oral paclitaxel hydrogel would facilitate effective and convenient treatment of various oral cancers with reduced toxicity. Therefore, the thermoreversible and mucoadhesive hydrogel, which has a sol-gel transition property, for oral paclitaxel application could have potential as a convenient and effective local delivery system. DMβCD may be a useful solubility enhancer for development of oral hydrogels containing poorly water-soluble drugs.
